# Procalcitonin for Diagnostics and Treatment Decisions in Pediatric Lower Respiratory Tract Infections

**DOI:** 10.3389/fped.2017.00183

**Published:** 2017-08-28

**Authors:** Philipp Baumann, Gurli Baer, Jessica Bonhoeffer, Aline Fuchs, Verena Gotta, Ulrich Heininger, Nicole Ritz, Gabor Szinnai, Jan Bonhoeffer

**Affiliations:** ^1^Department of Pediatric Infectious Diseases and Vaccinology, University of Basel Children’s Hospital, Basel, Switzerland; ^2^University of Basel Children’s Hospital, Basel, Switzerland; ^3^Department of Pediatric Pharmacology and Pharmacometrics, University of Basel Children’s Hospital, Basel, Switzerland; ^4^Department of Pediatric Endocrinology and Diabetology, University of Basel Children’s Hospital, Basel, Switzerland

**Keywords:** respiratory tract infections, pneumonia, procalcitonin, anti-bacterial agents, children

## Abstract

Mortality and morbidity remain high in pediatric lower respiratory tract infections (LRTIs) despite progress in research and implementation of global diagnostic and treatment strategies in the last decade. Still, 120 million annual episodes of pneumonia affect children younger than 5 years each year leading to 1.3 million fatalities with the major burden of disease carried by low- and middle-income countries (95%). The definition of pneumonia is still challenging. Traditional diagnostic measures (i.e., chest radiographs, C-reactive protein) are unable to distinguish viral and from bacterial etiology. As a result, common antibiotic overuse contributes to growing antibiotic resistance. We present an overview of current evidence from observational and randomized controlled trials on a procalcitonin (PCT)-based diagnosis of pediatric LRTIs and discuss the need for an adequate PCT threshold for antibiotic treatment decision-making.

## Morbidity and Mortality of Lower Respiratory Tract Infections (LRTIs) in Children

Community-acquired pneumonia (CAP) remains a leading cause of childhood morbidity and mortality worldwide. About 120 million episodes of pneumonia are estimated to affect children younger than 5 years each year with as many as 14 million episodes being severe enough to require hospital admission and 1.3 million episodes leading to death (1–3). CAP affects children younger than 2 years most frequently and most severely (2, 4). The majority of pediatric cases (95%) occur in low- and middle-income countries (e.g., incidence in Africa 0.27 episodes per child-year) with 80% of CAP fatalities occurring in south-east Asia and Africa (2). In contrast, the incidence in high-income countries is much lower. For example, Europe has a pneumonia incidence of 0.03 per child-year with 1.4% of the global mortality. However, pediatric respiratory tract infections still represent a significant part of each country’s childhood morbidity in both the developing and the developed world: in Nigeria, 44% of pediatric hospital admissions are due to LRTIs (5), whereas in continental Europe, 9% of admitted children present LRTI (6). Non-fatal morbidity and pneumonia severity are driven by complications including pleural effusions (from 1 to 16% of pediatric pneumonia cases admitted to EDs) (7–9), empyema (4%) (8), and necrotizing pneumonia (0.8%) (10).

## Diagnostic Dilemma and Antibiotic Overuse

Reliable differentiation between uncomplicated and self-limiting acute respiratory tract infections and more severe disease requiring antibiotic treatment remains challenging. Bacterial pneumonia in need of antibiotic treatment may complicate viral respiratory tract infection. However, the presence of bacteria in the respiratory tract may not require antibiotic treatment as the colonization of the upper respiratory tract does not necessarily lead to pneumonia and as an immunocompetent patient may clear the bacteria without antibiotic treatment. Therefore, the physician faces suboptimal diagnostic tools to make informed treatment decisions. The difficulty in diagnosing LRTIs in need of antibiotic treatment in a pediatric patient is based on two major issues. First, causative etiology is highly variable, and mixed viral–bacterial infections can occur in 30–90% of pneumonia cases in children (11–14). Blood culture results are not available immediately and rarely positive in the outpatient (<2%) (15), and organisms identified from nasopharyngeal swabs may only reflect colonization. Better correlation can be expected from endotracheal aspirates, but this stays reserved for ventilated intensive care patients or patients undergoing bronchial lavage. Second, biomarkers of inflammation and radiologic imaging have not been reliable so far in differentiating viral from bacterial infections and are not available in every medical setting. Therefore, only non-specific clinical parameters (tachypnea, chest indrawing in mild and moderate cases, reduced fluid uptake, and/or reduced consciousness in very severe cases) with questionable ability to distinguish viral from bacterial LRTI, and no diagnostic tests are included in the WHO definition of childhood pneumonia and in the British Thoracic Society guidelines for the management of uncomplicated pediatric pneumonia (16, 17). However, clinical parameters remain an important element of diagnosis and assessment of disease severity in every medical setting, including low- and medium-income countries.

Diagnostic shortcomings, lack of time, and physician’s uncertainty in the light of potential life-threatening complications seem to trigger antibiotic prescribing for pediatric childhood infections, and many prescriptions are made “just in case” (18, 19). The resulting antibiotic overtreatment of pediatric chest infections (20, 21) has to be seen critically in the light of increasing antibiotic consumption and resistance rates (22).

## Diagnostic Uncertainty of Laboratory and Radiological Tests

With the intention to avoid undertreatment or overtreatment of LRTI, acute phase reactants [e.g., C-reactive protein (CRP), white blood cell count], and radiographic tests are widely used for daily decision-making on antibiotic treatment, despite their known limitations. In a meta-analysis including 8 studies and 1,230 patients, only a relatively weak cumulative positive predictive value of 64% could be calculated for a serum CRP exceeding 40–60 mg/L (23). Korppi showed a specificity of 82% and a sensitivity of 34% for the combination of white blood cell count, CRP, procalcitonin (PCT), and erythrocyte sedimentation rate for the prediction of alveolar infiltrates on chest radiographs (24). A number of further studies aimed at increasing the predictive performance by combining various laboratory parameters, but no combination truly succeeded in the development of a convincing algorithm for differentiating CAP in need of antibiotic treatment from other febrile LRTI (11, 25–27). CRP-guided antibiotic treatment has not been tested against standard care in children so far. However, a recent meta-analysis for adults with acute respiratory tract infections demonstrated a moderate reduction of antibiotic prescribing when CRP was included in treatment decision-making [relative risk (RR) 0.7, 95% CI 0.60–0.90] (28). Further, chest radiographs are not reliable and therefore not recommended in non-complicated febrile LRTI, as numerous studies have shown high intrarater and interrater variabilities. A strikingly low interrater agreement of only 9% in non-alveolar pneumonia could be shown by Ben Shimol et al., even when standardized radiologic WHO criteria were used by all raters (29, 30).

## Procalcitonin

The shortcomings of traditional diagnostic measures have driven studies investigating PCT as a biomarker. PCT is a precursor peptide of calcitonin and is released as a part of the pro-inflammatory response of the innate immune system from parenchymal cells reaching detectable levels within 4 h after endotoxin stimulation, i.e., much earlier than CRP (31). High PCT (usually >2 ng/mL) seems to be good marker for blood culture-positive pneumococcal infection in CAP. Decrease of PCT with amoxicillin treatment is usually rapid, and persistence of high PCT levels is generally due to a pneumococcal pleural focus that antibiotics cannot reach (32–34). It was shown that moderately elevated PCT (1–2.5 ng/mL) could be found in CAP with positive blood culture for *Streptococcus pneumoniae* mainly in bacterial superinfection after an initial viral episode, possibly reflecting transitory immune impairment (33). Further, PCT has been shown to strongly correlate with sepsis and septic shock in neutropenic (35, 36) and immune-competent patients (32, 37). PCT is particularly good in differentiating bacterial from viral meningitis in children (38) and seems to be useful to rule-in pyelonephritis, but studies on urinary tract infections display a high heterogeneity, which is why PCT cannot be recommended for antibiotic management of urinary tract infections at this point. PCT seems to be a useful marker for reduction of the antibiotic use in adult and neonatal intensive care patients (39–42), whereas data available for pediatric patients in intensive care are rather inconclusive so far and is not based on randomized controlled trials (RCTs) (43).

## PCT-Guided RCTs in Adults

The first available retrospective and observational data on adult CAP motivated Christ-Crain et al. (44) to design a RCT testing PCT guidance in adult patients with all kinds of LRTI presenting to the emergency room. The authors used the available evidence at this point of time for the first definition of PCT treatment thresholds. Previous studies on chronic obstructive pulmonary disease and sepsis had shown that the presence of bacterial infections was highly unlikely with PCT values <0.1 ng/mL and likely with PCT ≥ 0.5 ng/mL, but an exact treatment threshold was unknown. Therefore, the authors implemented an interventional PCT treatment algorithm incorporating the increasing risk of bacterial infection with increasing PCT values: PCT < 0.1 ng/mL: bacterial infection highly unlikely and antibiotic treatment strongly discouraged, PCT < 0.25 ng/mL: bacterial infection unlikely and antibiotic treatment rather discouraged, PCT ≥ 0.25 ng/mL: bacterial infection possible and start of antibiotic treatment advised, PCT ≥ 0.5 ng/mL: bacterial infection suggestive and antibiotic treatment strongly advised. The control group was treated according to international guidelines. Reassessment was possible after 6–24 h. The PCT guidance resulted in a significant lower antibiotic prescription rate (RR 0.49, 95% CI 0.44–0.55, *p* < 0.0001) without compromising patients’ safety outcome (44).

Following this informative finding, in a second interventional trial with identical PCT guidance for CAP, Christ-Crain et al. (45) proved PCT to be useful not only for the reduction of antibiotic prescribing but also for shortening antibiotic exposure by the reassessment of patients on 4, 6, and 8 days following enrollment. Antibiotic exposure was significantly shortened by 55% (*p* < 0.001) in the PCT-guided group with similar patients’ safety outcome in both the intervention and the control groups (45).

The promising results of these first interventional studies were confirmed later in a large multicenter RCT involving 1,359 adult patients presenting to the EDs with any LRTI, in which the intervention group was treated with antibiotics according to the same PCT guidance algorithm and the control group was treated according to the international guidelines. The mean duration of antibiotic exposure was significantly shorter (5.7 vs. 8.7 days; relative change −34.8%; 95% CI −40.3% to −28.7%), and the antibiotic prescription rates of all LRTI were significantly lower (75.4 vs. 87.7%; −12.2%; −16.3 to −8.1%) in the intervention group without adverse effect on patients’ outcome (46).

## PCT-Guided RCTs in Children and Adolescents

The success of PCT guidance in adults with LRTI also triggered pediatric research in this field. Following observational trials in children with promising results (32, 33, 47, 48), two RCTs have been published until to date in the English language. Esposito et al. published the first RCT in hospitalized children (>1 month and <14 years of age) with pneumonia in 2011. Diagnosis of pneumonia was made based on clinical findings (fever or cough, tachypnea, dyspnea or respiratory distress, and breathing with grunting or wheezing with rales) and confirmed by chest radiography (infiltration or consolidation). Patients with pleural effusions, empyema, lung necrosis and pneumatocele, underlying chronic disease, malnutrition, and patients under antibiotic treatment were excluded. Patients were randomized according to the adult interventional studies and were allocated to PCT-guided antibiotic treatment or to treatment according to international guidelines. In the intervention group, children did not receive antibiotic treatment, if PCT values were <0.25 ng/mL and were treated with antibiotics, if PCT values were ≥0.25 ng/mL. PCT was measured every 2 days until hospital discharge. Three hundred and ten children were enrolled on hospitalization, and after hospital discharge, all children were reassessed at 14 (SD 2) and 28 (3) days following randomization. Fourteen percent of CAP cases in the intervention group did not have to be treated according to constant low PCT values, whereas all patients in the control group received antibiotic treatment. Most of PCT-guided patients had their antibiotic treatment stopped on day 6 (37.4%) or 8 (46.6%). All antibiotic treatment was stopped according to PCT guidance latest on day 10. In contrast, most antibiotic treatment periods in the control group lasted until day 10 (82.6%) with some treatment periods extended until day 12 (25.2%) or day 14 (13.5%). In line with the adult data, this trial demonstrated a significant reduction of both antibiotic treatment rate (85.8 vs. 100%, *p* < 0.05) and duration (5.4 vs. 11.0 days, *p* < 0.05) by PCT-guided treatment without compromising patients’ outcome: rates of disease relapse and secondary antibiotic prescriptions were similar in the intervention and the control groups. Further, antibiotic-related adverse events were lower in the PCT group (4 vs. 25%, *p* < 0.05) (49). This first RCT for PCT guidance in pediatric LRTI is very valuable for pediatricians and clinical researchers, because it showed for the first time the feasibility of PCT guidance in pediatric patients, using the PCT treatment threshold 0.25 ng/mL adopted from adult studies. Further, a moderate, but significant reduction of antibiotic treatment could be achieved. The trial is limited by including only uncomplicated hospitalized pneumonia cases, which were diagnosed by chest radiograph. Further, with complications excluded, safety assessment in severe and complicated cases was limited as also correctly stated by the authors.

In 2013, the authors of the present study published the second RCT involving 337 children and adolescents with any LRTI presenting to the pediatric ED of two tertiary care centers (50). Exclusion criteria were severe immune depression, immunosuppressive treatment, neutropenia, cystic fibrosis, acute laryngotracheitis, and hospital stay within previous 14 days. The treatment algorithm in the interventional PCT-guided group was the same as in the previous adult studies. It increasingly encouraged antibiotic treatment according to increasing PCT values: strongly discouraged (<0.1 mg/L), discouraged (0.1–0.25 ng/mL), encouraged (0.26–0.5 ng/mL), and strongly encouraged (>0.5 ng/mL). Patients and laboratory values were reassessed routinely on day 3 and 5 after enrollment and possibly earlier if patients appeared severely sick. On the last day of assessment (day 5), further antibiotic treatment duration was determined also according to PCT values: >1 ng/mL: 7 days, 0.51–1 ng/mL: 5 days, 0.26–0.5 ng/mL: 3 days, and <0.25 ng/mL: no antibiotic. The study did not demonstrate an overall reduced antibiotic prescription rate: PCT group 62% vs. control group 56%, *p* = 0.36. PCT guidance treated significantly more patients in the non-CAP subgroup (45% vs. 17%, *p* = 0.002), whereas in CAP, there was no difference (71% vs. 79%, *p* = 0.25). However, the duration of antibiotic exposure was reduced in the PCT-guided group in all LRTI cases (duration 4.5 vs. 6.3 days, *p* = 0.039) and in the CAP subgroup (5.7 vs. 9.1 days, respectively, *p* = 0.001). In contrast, in the subgroup of non-CAP LRTI, antibiotic treatment was significantly prolonged with PCT guidance (2.4 vs. 1.6 days, *p* = 0.01) (50). Patients’ safety outcome was not affected by PCT guidance: 23% in the PCT-guided group and 20% in the control group showed serious adverse events, complications of LRTI, or disease-specific failure (odds ratio 1.16, 95% CI 0.69–1.97). The most important result of this trial was the feasibility and safety confirmation of PCT guidance in pediatric LRTI cases, because in the PCT-guided intervention group, all antibiotic treatment decisions were based solely on PCT levels irrespective of clinical, laboratory, or radiographic findings. The drawbacks of PCT guidance were the failure to reduce antibiotic prescription in all LRTI, the higher treatment rate, and longer treatment duration in non-CAP LRTI. A potential explanation for this might be that the PCT treatment threshold previously tested in adults (0.25 ng/mL) was too low for the pediatric population. A sub-analysis of this study (Baumann et al., unpublished) demonstrated the PCT distribution in different radiologically diagnosed subgroups: non-CAP LRTI [*n* = 118, PCT median 0.2 ng/mL, interquartile range (IQR) 0.1–0.4], CAP (*n* = 154, 0.8, 0.2–5.3), and lobar pneumonia (*n* = 33, 8.0, 1.9–20.6), *p* < 0.05. On the basis of these results, we speculate that the adoption of the adult treatment threshold of 0.25 ng/mL was not adequate for children and that higher PCT treatment thresholds of possibly 0.5 or 1 ng/mL might be more appropriate. Median PCT levels in children and adolescents with any LRTI in this study were slightly higher than observed in the largest adult RCT [median: 0.28 ng/mL (IQR 0.14–2.17) vs. 0.24 (0.11–1.36)]. This may further suggest that, in general, children develop higher levels of PCT in response to inflammatory stimuli compared to adults. This is supported by a retrospective study published by Cohen et al. in 2012 investigating the performance of different PCT levels in predicting clinical responses to beta-lactam treatment in febrile pediatric CAP (34). A rapid response with apyrexia within 48 h as a proxy for pneumococcal infection was predicted with best accuracy by a high PCT ≥ 3 ng/mL. Children with a rapid response to antibiotic treatment, and therefore likeliness of pneumococcal infection, revealed significantly higher PCT levels than the ones with delayed response (i.e., >48 h) [median (IQR) 3.7 (1–9.4) vs. 0.7 (0.2–2.9), *p* = 0.002] (34).

Despite these first positive results, there are still limitations to PCT use. It has been shown than some few patients do not reveal high PCT values in case of local infection with negative blood culture or in severe systemic infections (51). Further, PCT might be elevated in case of viral or *Mycoplasma pneumoniae* infection (33, 52). It is likely that future studies evaluating higher PCT thresholds in pediatric respiratory tract infections may be able to show antibiotic-sparing effects, but may also reveal a decrease in negative predictive value. Further, a PCT higher than 1 or 2 ng/mL in suspected viral respiratory tract infection might be caused by bacterial superinfection. Thus, PCT use for antibiotic treatment decision-making in pediatric LRTI cannot replace, but serve as a useful element of comprehensive clinical decision-making.

An increasingly interesting role play modern rapid diagnostic tests such as multiplex polymerase chain reaction (mPCR). A recent retrospective cohort study with 4,779 hospitalized pediatric patients showed a reduction of antibiotic treatment duration when patients managed with mPCR were compared to a historic cohort, in which mPCR has not been used (median 4 vs. 5 days, *p* < 0.01). This held true even though the mPCR group appeared to be more severely sick, and patients from that group were admitted to the intensive care unit more often (53). Quantitative real-time PCR of *S. pneumoniae* load in nasopharyngeal secretions in combination with rapid immunofluorescence testing for viruses in suspected mixed viral–bacterial LRTIs have the potential to further facilitate the diagnosis of bacterial CAP (54) and the determination of antibiotic treatment need, when they are integrated in treatment decision models alongside with clinical and laboratory parameters.

## Conclusion

Over the past 15 years, pediatric evidence emerged for PCT as a useful diagnostic component for antibiotic treatment decisions in febrile LRTI and for other infectious diseases such as sepsis, meningitis, and urinary tract infections. Feasibility and safety of PCT antibiotic guidance were proven for adult thresholds used in children and adolescents with LRTIs irrespective of other diagnostic tests. Still, the number of pediatric studies and participants is limited, and the treatment threshold for pediatric use remains to be confirmed. The currently published low treatment thresholds for pediatric LRTI may be an important reason for the moderate effect of PCT guidance on antibiotic sparing so far. Based on the available literature to date, large high-quality studies testing higher PCT treatment thresholds and possibly novel point of care tests would be informative. The integration of rapid viral immunofluorescence tests and bacterial real-time mPCR could further help to individualize antibiotic treatment of pediatric LRTI. PCT can be a useful component of a comprehensive clinical assessment and supports treatment decisions in pediatric LRTI.

## Author Contributions

PB, GB, JeB, AF, VG, UH, NR, GS, and JaB contributed to the conception and drafting or revision process of the article and approved the final manuscript. All authors are accountable for all aspects of the manuscript.

## Funding Statement

The authors received no specific funding for the present work. For the ProPAED study (50), BRAHMS previously provided PCT test kits and platform to the authors. BRAHMS had no role neither in the published ProPAED study nor in the design or preparation of this manuscript.

## Conflict of Interest Statement

The authors declare that the research was conducted in the absence of any commercial or financial relationships that could be construed as a potential conflict of interest. The reviewer, JR, and handling editor declared their shared affiliation.
